# Factors Associated with Tuberculosis and Rifampicin-Resistant Tuberculosis amongst Symptomatic Patients in India: A Retrospective Analysis

**DOI:** 10.1371/journal.pone.0150054

**Published:** 2016-02-26

**Authors:** Sreenivas Achuthan Nair, Neeraj Raizada, Kuldeep Singh Sachdeva, Claudia Denkinger, Samuel Schumacher, Puneet Dewan, Shubhangi Kulsange, Catharina Boehme, Chinnambedu Nainarappan Paramsivan, Nimalan Arinaminpathy

**Affiliations:** 1 World Health Organization, Country Office for India, New Delhi, India; 2 Foundation for Innovative New Diagnostics, New Delhi, India; 3 Central TB Division, Government of India, New Delhi, India; 4 Bill and Melinda Gates Foundation, New Delhi, India; 5 Department of Infectious Disease Epidemiology, Imperial College London, London, United Kingdom; Public Health Research Institute at RBHS, UNITED STATES

## Abstract

**Background:**

Tuberculosis remains a major public health challenge for India. Various studies have documented different levels of TB and multi-drug resistant (MDR) TB among diverse groups of the population. In view of renewed targets set under the End TB strategy by 2035, there is an urgent need for TB diagnosis to be strengthened. Drawing on data from a recent, multisite study, we address key questions for TB diagnosis amongst symptomatics presenting for care: are there subgroups of patients that are more likely than others, to be positive for TB? In turn, amongst these positive cases, are there factors—apart from treatment history—that may be predictive for multi-drug resistance?

**Methods:**

We used data from a multi-centric prospective demonstration study, conducted from March 2012 to December 2013 in 18 sub-district level TB programme units (TUs) in India and covering a population of 8.8 million. In place of standard diagnostic tests, upfront Xpert MTB/RIF testing was offered to all presumptive TB symptomatics. Here, using data from this study, we used logistic regression to identify association between risk factors and TB and Rifampicin-Resistant TB among symptomatics enrolled in the study.

**Results:**

We find that male gender; history of TB treatment; and adult age compared with either children or the elderly are risk factors associated with high TB detection amongst symptomatics, across the TUs. While treatment history is found be a significant risk factor for rifampicin-resistant TB, elderly (65+ yrs) people have significantly lower risk than other age groups. However, pediatric TB cases have no less risk of rifampicin resistance as compared with adults (OR 1.23 (95% C.I. 0.85–1.76)). Similarly, risk of rifampicin resistance among both the genders was the same. These patterns applied across the study sites involved. Notably in Mumbai, amongst those patients with microbiological confirmation of TB, female patients showed a higher risk of having MDR-TB than male patients.

**Conclusion:**

Our results cast fresh light on the characteristics of symptomatics presenting for care who are most likely to be microbiologically positive for TB, and for rifampicin resistance. The challenges posed by TB control are complex and multifactorial: evidence from diverse sources, including retrospective studies such as that addressed here, can be invaluable in informing future strategies to accelerate declines in TB burden.

## Background

Tuberculosis (TB) is one of the world’s deadliest communicable diseases. Of the estimated 9.6 million new TB cases in 2014, India alone accounted for 23% of total cases [1]. While most cases of TB are curable with cost-effective combination chemotherapy, multi-drug-resistant (MDR) TB is becoming an increasing challenge, accounting for an estimated 480,000 cases globally in 2014, of whom only 123,000 were detected. Treatment for MDR-TB is costly and protracted, and shows substantially lower success rates than for drug-sensitive TB. In India alone there are an estimated 64,000 MDR-TB cases occurring annually among notified pulmonary TB cases [2]. Drug resistance surveys in several states suggest that the prevalence of MDR TB in India is 2–3 percent among new cases and 12–17 percent among reinfection cases [3]. Although TB and MDR TB detection and notification rates have seen recent improvements, to achieve the global target set under the End TB strategy [4], there is a need to intensify case detection of TB and drug-resistant TB.

Typically in India and elsewhere, patients referred to the national TB programme for diagnosis are those showing symptoms that are suggestive, but not necessarily definitive, for TB disease (symptoms such as chronic cough, fever and weight loss). These patients, ‘presumptive TB symptomatics’, are offered microbiological testing for TB, most often using smear microscopy: an affordable and well-established diagnosis, that nonetheless can miss upto half of TB cases. Detection of drug resistance offers additional challenges, being a resource- and time- intensive procedure that is currently offered only to specific risk groups, primarily those with a history of TB treatment. Given such limitations, therefore, there has been considerable uncertainty around the predictors of TB and MDR-TB in the symptomatic population. That is: are there subgroups amongst presumptive TB symptomatics that are more likely than others, to be positive for TB? In turn, amongst these positive cases, are there factors—apart from treatment history—that may be predictive for multi-drug resistance?

In the present study we aim to address these questions by taking advantage of a recent large, multi-site study in India where presumptive TB symptomatics were offered high-sensitivity, rapid molecular testing, in place of existing diagnostic tests. Casting fresh light on TB and MDR-TB cases in the symptomatic population,that might otherwise have been missed by smear microscopy, this data provides a unique opportunity for identifying patient factors to guide prioritization of diagnosis efforts. Moreover, previously unrecognised patient factors may merit further investigation, for their potential epidemiological importance.

## Methods

We drew from a recent multi-centric prospective demonstration study conducted by FIND (Foundation for Innovative New Diagnostics), which enrolled more than 100,000 presumptive TB symptomatics, under programmatic conditions, across 18 diverse settings from March’12 to December’13 [5]. In place of existing diagnostic tests, upfront Xpert MTB/RIF testing was provided to all TB symptomatics seeking care; high levels of TB and MDR TB were reported [5]. The 18 sites were purposively selected based on the availability of free treatment for patients diagnosed with rifampicin resistance, and to represent diverse geographic and demographic settings across the country. Of these, 8 sites were in rural areas catering to a population of 3.9 million, 6 sites were in urban areas catering to a population of 3.4 million and 4 sites were in tribal and hilly area covering a population of 1.5 million. Altogether, these 18 sites account for 8.8 million people having access to TB diagnostic services. As in any programmatic setting in India, presumptive TB symptomatics were referred by a range of providers, together representing a range of healthcare settings from primary care to inpatient settings. Further details on the study design are given elsewhere [5].

The study protocol was approved by the Institution Ethics Committee of the National Tuberculosis Institute, Bangalore, India. Structured informed consent forms were used for obtaining written consent from all subjects enrolled in the study. Before taking consent, patients were informed about the study in vernacular language by the trained staff. For illiterate patients, consent was taken in presence of literate witness; similarly written consent for the children less than 18 years of age was obtained from the parents / guardians accompanying them. Approval for the study was granted by the Central TB Division, Ministry of Health and Family Welfare, Government of India.

In the present work, data analysis was performed using logistic regression to identify factors associated with two separate outcomes: (i) bacteriologically positive TB (i.e. Xpert positive) amongst all cases receiving a GeneXpert test, and (ii) Rifampicin resistance (again as diagnosed by Xpert), amongst all patients testing positive for TB. We considered five covariates from the available data that were specified prior to conducting the analysis: gender (Male or Female); Age (upto 15 yrs; 16–64 yrs and 65 yrs+); history of prior TB treatment (yes or no); the type of provider (public sector; private provider; medical college; non- governmental organizations (NGO); and anti-retroviral treatment (ART) centre), and the geographical area (rural, urban, and tribal or hilly). In particular, denoting these patient characteristics respectively as *X*_*Gender*_, *X*_*Age*_, *X*_*History*_, *X*_*Provider*_ *and X*_*Area*_, we fitted the model:

$$logit\;Y_{TB}=\;\beta_{0}+\beta_{Gender}\;X_{Gender}+\beta_{Age}X_{Age}+\beta_{History}X_{History}+\beta_{Provider}X_{Provider}+\beta_{Area}X_{Area},$$

where *Y*_*TB*_ is the probability of a symptomatic patient being positive for TB. Amongst those patients testing positive for TB, we similarly fitted the model:

$$logit\;Y_{MDR\;|\;TB}=\beta_{0}+\;\beta_{Gender}\;X_{Gender}+\beta_{Age}X_{Age}+\beta_{History}X_{History}+\beta_{Provider}X_{Provider}+\beta_{Area}X_{Area},$$

where *Y*_*MDR | TB*_ is the probability of a confirmed TB case having MDR-TB. Because Mumbai (an urban site in Maharashtra state) has a unique TB epidemic [6–7], with high levels to rifampicin resistant reported and also observed in our dataset, we stratified the analysis to consider Mumbai data separately from the other locations. All analyses were performed in the statistical analysis software R.

## Results

Under the study, a total of 1, 04, 276 presumptive TB cases were enrolled, of which 22,686 (21.8%) were diagnosed with TB and out of them 2,765 (2.7%) were found to be resistant to rifampicin. (Table 1)

**Table 1:** Demographic profile of study participants.

	N	% (of N)	TB	% (of row)	Rif- Resistance	% (of row)
**Total**	1,04,276	100	22,686	21.8%	2,765	2.7%
**Gender**						
Female	38,384	36.8	6,686	17.4%	1,027	2.7%
Male	65,892	63.2	16,000	24.3%	1,738	2.6%
**Past history of anti TB treatment**						
Previous TB	20,615	19.8	8,125	39.4%	1,912	9.3%
New	83,255	79.8	14,561	17.5%	853	1.0%
**Age category (Years)**						
Child	4,647	4.5	514	11.1%	91	2.0%
Adult	80,655	77.3	19,137	23.7%	2,490	3.1%
Elderly	18,974	18.2	3,035	16.0%	184	1.0%
**Provider**						
Public	78,740	75.5	16,591	21.1%	1,443	1.8%
Private	2,738	2.6	757	27.6%	138	5.0%
Med. college	16,453	15.8	4,273	26.0%	1,102	6.7%
NGO	4,903	4.7	815	16.6%	62	1.3%
ART centre	1,442	1.4	250	17.3%	20	1.4%
**Settings**						
Mumbai	12,041	11.5	3,454	28.7%	1,233	10.2%
Urban (excl. Mumbai)	36,787	35.3	8,173	22.2%	806	2.2%
Rural	40,832	39.2	7,964	19.5%	496	1.2%
Tribal/hilly	14,616	14.0	3,095	21.2%	230	1.6%

Table 2 shows the results of the multivariable analysis, for factors associated with bacteriologically positive TB case detection. Risk factors that are consistent across the TUs include: male gender; history of TB treatment; adult age compared with either children or the elderly; type of provider, and geographical location. Importantly, TB symptomatics referred from ART centers had a lower rate of TB detection compared to patients referred by the public sector. Moreover, in Mumbai results suggest a higher rate of bacteriologically positive TB amongst patients referred by private providers, medical colleges and NGOs, compared to those referred by the public sector, while in other TUs, there is a higher rate amongst patients referred by private providers, medical colleges and ART centres.

**Table 2:** Predictors of TB positivity amongst all TB symptomatics. Owing to the size of the dataset, all associations shown as significant have p < 10^−5^, with the exception of Mumbai patients referred by ART centres (*p* = 0.01).

Predictor	Levels	Odds ratio, unadjusted (95% C.I.)	Odds ratio, adjusted (95% C.I.)
**Mumbai TU**			
Sex	Female	0.84 (0.77, 0.91)	0.85 (0.79, 0.93)
	Male	Reference	Reference
HXTB	Previous history	2.4 (2.22, 2.61)	2.22 (2.05, 2.42)
	No previous history	Reference	Reference
Age	Child	0.42 (0.35, 0.5)	0.49 (0.4, 0.59)
	Adult	Reference	Reference
	Elderly	0.56 (0.48, 0.65)	0.56 (0.48, 0.65)
Provider	Private	2.19 (1.85, 2.59)	1.95 (1.65, 2.32)
	Medical College	1.52 (1.4, 1.66)	1.32 (1.21, 1.43)
	NGO	1.51 (1.1, 2.06)	1.57 (1.13, 2.16)
	ART Centre	0.59 (0.32, 1.01)	0.47 (0.25, 0.82)
	Public	Reference	Reference
**Other TUs**			
Sex	Female	0.61 (0.58, 0.63)	0.62 (0.6, 0.65)
	Male	Reference	Reference
HXTB	Previous history	3.06 (2.95, 3.18)	2.92 (2.81, 3.03)
	No previous history	Reference	Reference
Age	Child	0.37 (0.33, 0.41)	0.43 (0.38, 0.48)
	Adult	Reference	Reference
	Elderly	0.63 (0.61, 0.66)	0.6 (0.57, 0.63)
Provider	Private	1.3 (1.17, 1.45)	1.38 (1.23, 1.53)
	Medical College	1.19 (1.13, 1.25)	1.11 (1.06, 1.17)
	NGO	0.76 (0.71, 0.83)	0.94 (0.87, 1.02)
	ART Centre	0.79 (0.68, 0.9)	0.69 (0.59, 0.79)
	Public	Reference	Reference
Area	Tribal & Hilly	1.15 (1.1, 1.2)	1.11 (1.06, 1.17)
	Urban	1.2 (1.16, 1.25)	1.07 (1.03, 1.11)
	Rural	Reference	Reference

Table 3 shows results for factors associated with rifampicin resistance, amongst diagnosed TB cases. As would be expected, treatment history is a significant risk factor for rifampicin resistance, both in Mumbai and in other TUs. While the elderly (65+ yrs) have significantly lower risk than other age groups, there is no evidence that pediatric TB cases have any smaller risk of rifampicin resistance than adults, in any of the sites in this study (OR 1.23 (95% C.I. 0.85–1.76)).

**Table 3:** Risk groups for Rifampicin resistance amongst Xpert positive cases. As in Table 2, all significant associations have p < 10^−5^, with the exceptions of: Mumbai patients referred by the private sector (*p* = 0.01) and by NGOs (*p* = 0.001).

Predictor	Levels	Odds ratio, unadjusted (95% C.I.)	Odds ratio, adjusted (95% C.I.)
**Mumbai TU**			
Sex	Female	1.53 (1.33, 1.76)	1.5 (1.29, 1.74)
	Male	Reference	Reference
HXTB	Previous history	2.86 (2.48, 3.32)	2.85 (2.46, 3.31)
	No previous history	Reference	Reference
Age	Child	1.38 (0.98, 1.93)	1.23 (0.85, 1.76)
	Adult	Reference	Reference
	Elderly	0.65 (0.47, 0.87)	0.65 (0.48, 0.89)
Provider	Private	1.4 (1.06, 1.85)	1.44 (1.07, 1.91)
	Medical College	2.03 (1.74, 2.36)	1.82 (1.56, 2.13)
	NGO	2.54 (1.5, 4.27)	2.41 (1.4, 4.13)
	ART Centre	0.45 (0.07, 1.67)	0.35 (0.05, 1.34)
	Public	Reference	Reference
**Other TUs**			
Sex	Female	1.06 (0.95, 1.19)	1.12 (0.99, 1.26)
	Male	Reference	Reference
HXTB	Previous history	5.49 (4.91, 6.16)	5.6 (4.99, 6.3)
	No previous history	Reference	Reference
Age	Child	0.9 (0.6, 1.3)	0.95 (0.62, 1.4)
	Adult	Reference	Reference
	Elderly	0.49 (0.4, 0.59)	0.54 (0.44, 0.65)
Provider	Private	1.4 (1.02, 1.89)	1.89 (1.36, 2.58)
	Medical College	2.23 (1.96, 2.53)	2.26 (1.96, 2.59)
	NGO	0.61 (0.42, 0.85)	0.93 (0.63, 1.32)
	ART Centre	1.09 (0.65, 1.73)	1.32 (0.78, 2.12)
	Public	Reference	Reference
Area	Tribal & Hilly	1.21 (1.03, 1.42)	1.18 (0.99, 1.39)
	Urban	1.65 (1.47, 1.85)	1.08 (0.95, 1.23)
	Rural	Reference	Reference

Results also suggest notable risk patterns by sex: in sites other than Mumbai, female TB cases have no significant difference in rates of rifampicin resistance compared to male TB patients. In Mumbai, by contrast, female TB cases are significantly *more* at risk of rifampicin resistance than male cases. As this result arises after adjusting for age, treatment history, and referring provider in the multivariable analysis, it is not necessarily due, for example, to higher levels of treatment history amongst females. Indeed, regressing treatment history against sex suggested a lower rate of treatment history amongst female vs male cases in Mumbai (odds ratio 0.89 (95% CI 0.84–0.93)).

Finally, there appears again to be a role for the treating provider, with TB cases being catered by private providers and medical colleges having a greater risk of being rifampicin-resistant, across all TUs, than those referred by the public sector.

## Discussion

To meet the renewed global targets in TB control, there is a pressing need for a better understanding of specific groups that bear a disproportionate TB burden. Here we have taken advantage of findings from a recent, large-scale GeneXpert demonstration study, to explore patient characteristics associated with diagnosis for TB and for Rifampicin resistance. While this work cannot by itself offer conclusive evidence for causal associations, results could point the way for further, in-depth analysis wherever hitherto unexpected associations emerge. Such analysis could provide a first step, either in identifying specific groups for targeting in future case-finding, or in determining risk factors that may be amenable to intervention.

Several factors identified by this analysis are consistent with what is already understood about TB burden: for example, higher TB diagnosis rates in male than female TB symptomatics being in agreement with the higher prevalence of TB in men [8]. Moreover, a lower risk of TB and MDR-TB amongst the elderly, compared to other age groups, may be due to reduced survival amongst those with TB infection [9].

However, some findings in particular suggest hitherto unrecognized risk factors. First, results (Table 3) suggest that rates of drug resistance amongst bacteriologically positive, pediatric cases are as high as in adults, across all of the sites involved. Owing to the challenges around bacteriological confirmation of pediatric TB, a major proportion of childhood TB cases are diagnosed clinically and initiated on first line TB treatment [10–11]. This makes laboratory diagnosis of drug-resistance in the pediatric population quite challenging. Our analysis suggests a need for strengthened drug-resistance surveillance in pediatric patients diagnosed with TB and/ or potentially reviewing the diagnostic and treatment strategy in presumptive pediatric TB. Second, despite a higher TB prevalence in males than females, TB cases of both genders show, overall, a similar risk of having MDR-TB. Indeed, in Mumbai the risk is greater amongst females than males, even when adjusting for treatment history, age and other factors. Risks of active TB among infected people are similar in both sexes in scenarios where the transmission rates are high [12–15]. A higher proportion of rifampicin resistance in female cases in Mumbai could point towards higher transmission of rifampicin resistance in that population. Although it remains unclear why this appears to be specific to Mumbai, the TB epidemic in Mumbai stands apart in other respects too. The city contains the largest slum in the world, and available evidence points to an MDR-TB epidemic in Mumbai that is more extensive than anywhere else in the country [6–7]. There is increasing recognition for the need to identify the key drivers behind this epidemic [16] such efforts may also cast valuable light on the patterns observed in the current study.

Our results also suggest some associations with respect to the referring provider: for example, Over 27% of HIV-positive TB symptomatics in this population were bacteriologically positive for TB (higher than 13% amongst smear-positive symptomatics) [17]. In the present analysis, however, the odds of TB detection amongst patients referred from ART centres is lower than those referred by the public sector. A potential explanation is that ART centres may simply be referring symptomatics more readily than in the public sector. Conversely, selective referral may explain why patients referred from the private sector and medical colleges were more likely to be diagnosed with TB than those from the public sector. Rifampicin resistance is higher amongst patients referred by the private sector than those referred by the public sector, perhaps suggestive of lower treatment quality in the private sector [18–19]. However, medical colleges also show higher rates of resistance: with quality of care in this sector likely to be higher than amongst private providers, a potential explanation is that medical colleges may tend to receive TB cases who have not responded to treatment elsewhere. Unfortunately it was not possible, with the available data, to explore further the potential role of these risk factors by provider.

Our study has other limitations to note. First, a true prevalence survey would aim to detect cases in the community, including those that have not sought care. Our analysis, by contrast, is based on TB symptomatics as a study population. Our results are therefore relevant to risk factors amongst presumptive TB symptomatics, as opposed to factors in the general population. Nonetheless, they cast light on potentially important factors for case finding in the general population,that merit further investigation. Secondly, while we adjusted in our analysis for key variables, residual confounding may remain. As such, any associations identified by this analysis should not be interpreted as causal analysis. More in-depth investigation is needed to explain the phenomenon of female TB cases in Mumbai having higher rates of drug resistance than male TB cases. Likewise, the role of the referring provider is an important subject for future work.

## Conclusion

In summary, the challenges posed by TB control are complex and multifactorial: evidence from diverse sources, including retrospective studies such as that addressed here, can be invaluable in informing future strategies to accelerate declines in TB burden.
